# Impacts of Enriching Growing Rabbit Diets with *Chlorella vulgaris* Microalgae on Growth, Blood Variables, Carcass Traits, Immunological and Antioxidant Indices

**DOI:** 10.3390/ani9100788

**Published:** 2019-10-11

**Authors:** Sameh A. Abdelnour, Asmaa M. Sheiha, Ayman E. Taha, Ayman A. Swelum, Saud Alarifi, Saad Alkahtani, Daoud Ali, Gadah AlBasher, Rafa Almeer, Fawaz Falodah, Bader Almutairi, Mohamed M. Abdel-Daim, Mohamed E. Abd El-Hack, Ismail E. Ismail

**Affiliations:** 1 Department of Animal Production, Faculty of Agriculture, Zagazig University, Zagazig 44511, Egypt; samehtimor86@gmail.com (S.A.A.); asmaasheiha76@gmail.com (A.M.S.); 2 Department of Animal Husbandry and Animal Wealth Development, Faculty of Veterinary Medicine, Alexandria University, Edfina 22758, Egypt; Ayman.Taha@alexu.edu.eg; 3 Department of Theriogenology, Faculty of Veterinary Medicine, Zagazig University, Zagazig 44511, Egypt; aymanswelum@zu.edu.eg; 4 Department of Zoology, College of Science, King Saud University, Riyadh 11461, Saudi Arabia; salarifi@ksu.edu.sa (S.A.); salkahtani@ksu.edu.sa (S.A.); aalidaoud@ksu.edu.sa (D.A.); galbeshr@ksu.edu.sa (G.A.); ralmeer@ksu.edu.sa (R.A.); ffalodah@ksu.edu.sa (F.F.); bomotairi@ksu.edu.sa (B.A.); 5 Pharmacology Department, Faculty of Veterinary Medicine, Suez Canal University, Ismailia 41522, Egypt; 6 Poultry Department, Faculty of Agriculture, Zagazig University, Zagazig 44511, Egypt; dr_ismailattia@yahoo.com

**Keywords:** rabbits, *Chlorella vulgaris*, growth, immunity, antioxidants

## Abstract

**Simple Summary:**

Using *Chlorella vulgaris* (CLV) as an immunomodulatory agent and antimicrobial activity to promote the immunity system has been reported in some previous studies. Our work investigated the impacts of dietary CLV supplementation on growing rabbit diets in terms of growth performance, carcass traits, hematobiochemical variables, immunity responses, and antioxidant status. Results showed that the CLV supplementation can positively affect the health status aspects of growing rabbit.

**Abstract:**

This work aimed to explore the effects of dietary supplementation of *Chlorella vulgaris* (CLV) on the growth performance, carcass traits, hematobiochemical variables, immunity responses, and the antioxidant status of growing rabbits. A total number of 100 rabbits were randomly distributed into four treatment groups, each of five replicates (25 rabbits/group). The experimental groups were as follows; control: a basal diet without supplementation, CLV0.5: basal diet + 0.5 g chlorella powder/kg diet; CLV1.0: basal diet + 1.0 g chlorella powder/kg diet, CLV1.5: basal diet + 1.5 g chlorella powder/kg diet. Live body weight (LBW), cumulative body weight gain (CBWG), feed intake (FI), and feed conversion ratio (FCR) were not affected by dietary CLV supplementation. Platelet count (PLT), hematocrit (HCT), means corpuscular hemoglobin (MCH), and mean corpuscular hemoglobin concentration (MCHC) values were significantly increased in the CLV0.5 group compared with the other treatment groups. Dietary supplementation of CLV (1.5 g/kg diet) significantly reduced the alanine aminotransferase (ALT) activity. The concentrations of serum triglycerides and very low-density lipoprotein (VLDL) were lower (*p <* 0.05) in the CLV-treated groups than those of the control. Supplemental CLV at all experimental levels gave the best values of immunoglobulins (IgG and IgM) and glutathione activities. Malondialdehyde (MDA) levels were lower in the animals that received CLV in their diet than those of the control group. Dietary supplementation of 1.0 g CLV/kg had the potential to enhance immune responses and antioxidant status, as well as reduce blood lipid accumulation. Therefore, it could be concluded that CLV supplementation to growing rabbit diets can improve the health status.

## 1. Introduction

In the extensive rabbit production, the time near weaning is the most critical period, when rabbits are extremely sensitive to multifactorial digestive disorders and face a theatrical life amendment in the food source, the immune system, as well as social status and environmental stressors [1,2]. These changes might have a serious economic loss on rabbit meat production. Researchers have demonstrated that the favorable impacts of using natural growth promoters such as probiotics, prebiotics, and phytogenic feed additives in rabbit diets can promote growth performance, feed efficiency, and immunity responses, as well as minimize high mortality and morbidity rates, especially during the weaning period [1,3].

In light of this, microalgae supplements have recently occupied a privileged place as a sustainable alternative resource to fatty fish (especially n3 and n6 fatty acids) to enhance the animal productivity [4,5]. In addition, it has been reported that the inclusion of microalgae in animal feeds enhanced growth performance, immunity, and antioxidants indices and meat quality in poultry, rodent, pigs, and ruminants [4,5]. *Chlorella vulgaris* is a kind of single-cell green algae that contains a diversity of a mixture of biological compounds such as protein, fats, carbohydrates vitamins, and bioactive compounds, such as polysaccharides, phenolic and volatile constituents, natural pigments, and sterols [6]. Furthermore, it is a rich source of polyunsaturated fatty acids (PUFAs), mainly γ-linolenic acid (CLA), eicosapentaenoic acid (EPA), docosahexaenoic acid (DHA), and arachidonic acid, which may be beneficial to animal and human health [7,8,9].

The use of chlorella as an immunomodulatory and antimicrobial agent to promote the immunity system in a broiler has been reported [7,8]. Moreover, Tsiplakou et al. [10] reported that the CLV inclusion in goat’s diet enhanced the immune responses and antioxidant indices. In addition, a significant improvement in the phagocytic activity of broiler chicken leukocytes was reported in response to feed enrichment with chlorella [11]. Likewise, An et al. [9] showed that chick feed having 0.15% dried CLV powder significantly enhanced growth performance, blood cell counts, and reduced the total lipids in serum compared to the unsupplemented group. Together, CLV has been revealed to enhance immune indices in rodents via its capability to boost the synthesis of cytokines such as IL-2 and IL-4 [12], stimulate phagocytic activity in poultry [11], and play a critical role in modern cancer therapy. Until now, it’s not clearly understood how the inclusion of CLV promotes the growth performance and immunity responses in rabbit.

To the best of our knowledge, there are no previous studies about the influences of dietary supplementation of CLV to rabbit diets. So, the current work examined the influences of dietary CLV supplementation on growth performance, carcass traits, hematobiochemical variables, and immunity and antioxidant indices of growing rabbits.

## 2. Materials and Methods

### 2.1. Ethical Statement

The experiment was carried out at the Rabbit Research Unit, Faculty of Agriculture, Zagazig University, Egypt. All protocols and experiments of the study were performed in accordance with the Local Experimental Animal Care Committee, and the ethics were permitted by the Institutional Committee of Animal Production, Faculty of Agriculture, Zagazig University, Zagazig, Egypt.

### 2.2. Experimental Design, Animals, and Management

A total of 100 healthy weaning male New Zealand white rabbits (5 weeks old, 635.71 *±* 2.84 g), were used in the current study. The experiment lasted for 8 weeks. Animals were randomly distributed into four experimental groups, each of five replicates (each replicate had five rabbits). The experimental groups were as follows; control: a basal diet without supplementation, CLV0.5: basal diet + 0.5 g chlorella powder/kg diet; CLV1.0: basal diet + 1.0 g chlorella powder/kg diet, and CLV1.5: basal diet + 1.5 g chlorella powder/kg diet. The *Chlorella vulgaris* was purchased and identified from Algal Biotechnology Unit, National Research Centre, 33 Bohouth St. Dokki, Giza, Egypt. Growing rabbits were reared in the rabbit research unit in wire single cages (30 cm width × 40 cm high × 50 cm length). Rabbits were fed ad libitum, and drinking water was automatically presented all time. The rabbits used in this experiment were raised under the same environmental, sanitary and management circumstances. Basal diet was formulated in accordance with the alimentary rations of the NRC [13] (Table 1). All verified diets were framed, pelleted, and kept at the farm during the trial.

### 2.3. Growth Performance

Feed intake (FI) was considered by subtracting the weight of the remained from the basic offered food. Daily body weight gain (DBWG), live body weight (LBW), and feed conversion ratio (FCR) were also determined cumulatively through the collected data by period.

### 2.4. Blood Hematology and Serum Metabolites

At the end of the trial, blood samples were aseptically gathered from the slaughtered rabbits (n = 5, for each group) and placed in sterile tubes. The hematological variables were assessed in accordance with Schalm [14]. In the complete blood samples, evaluations of red blood cells (RBCs), packed cell volume (PCV), hemoglobin (Hb), mean corpuscular volume (MCV), mean corpuscular hemoglobin concentration (MCHC), total leucocytes, basophils (BASO), eosinophils (ESIN), lymphocytes (LYM), and monocytes (MON) were analyzed using a Hema Screen18 automated hematology analyzer (Hospitex Diagnostics, Sesto Fiorentino, Italy). Another tube (sterile) was used for each animal for the assessment of serum metabolites; then, it was permitted to coagulate at room temperature (for 30 min), and next centrifuged for 15 min at 3500 rpm for serum separation. The serum samples were preserved at −20 °C until exploration.

The serum content of total protein, albumin, globulin, total cholesterol, triglycerides, uric acid, total and direct bilirubin, low-density lipoprotein (LDL), high-density lipoprotein (HDL), aspartate aminotransferase (AST), alanine amino transferase (ALT) and lactate dehydrogenase (LDH) were spectrophotometrically analyzed using commercial diagnostic kits provided from Biodiagnostic Co. (Giza, Egypt).

### 2.5. Antioxidants and Immunological Assays

Immunoglobulins (IgG and IgM) were measured based on the method termed by Akiba et al. [15]. The activities of antioxidants parameters were measured including: superoxide dismutase (SOD), reduced glutathione (GSH), and catalase (CAT) as well as malondialdehyde (MDA) (as a lipid peroxidation marker) by spectrophotometric methods (Hitachi spectrophotometer, Japan) using commercial biodiagnostic kits provided from BioMérieux (Marcy l’Etoile, France) according to the manufacturer’s instructions.

### 2.6. The Carcass Traits

By the end of the experiment (13 weeks old), five rabbits per group were randomly chosen, weighted, and exsanguinated after 12-h feed deprivation. Then, the carcasses were prepared for examination by means of eliminating skin, feet, paws, urinary bladder, alimentary, and genital organs. The hot carcass parts (main body, giblets, heart, kidneys, lungs, liver, and spleen) were weighted, itemized, and specified as g/kg of pre-slaughter weight and calculated as a relative weight from hot body weight. Carcass percentage = carcass weight × 100/LBW. Dressing percentage = (the carcass weight + the giblets weight) × 100/LBW.

### 2.7. Statistics

All the statistical analyses of the obtained results were achieved using the SAS software program [16]. The performance, hematobiochemical variables, antioxidants, and immunity status of the carcasses parameters were evaluated with a one-way analysis of variance (with the diet as the fixed factor) using the post-hoc Newman–Keuls test. The *p* < 0.05 was considered to be statistically significant.

## 3. Results

### 3.1. Growth Performance

Results of the impact of dietary supplementation of CLV on live body weight and the body weight gain of growing NZW rabbits, feed intake, feed conversion ratio, and carcass traits are presented in Table 2, Table 3 and Table 4. Supplementing the rabbit diets with CLV did not induce significant differences (*p* > 0.05) in live body weight (LBW) and cumulative body weight gain (CBWG); feed conversion ratio (FCR) and feed intake (FI); and carcass traits (dressing percentage, giblets, heart, kidney, lung, and liver) as compared to the control animals.

### 3.2. Blood Hematology

The impacts of adding CLV to the diets of growing rabbits on the erythrogram and leukogram counts are shown in Table 5. Significant variances (*p* < 0.05) in the all blood hematology traits were detected except for hemoglobin (HGB), red blood cells (RBCs), basophils (BASO), and eosinophils (ESIN) as a result to dietary CLV supplementation. The experimental group CLV1.5 showed significant white blood cells (WBC) count compared with those other CLV treated and control groups. Meanwhile, CLV0.5 had significantly higher PLT, HCT, MCH, and MCHC compared to the control and the other CLV treated rabbits. On the other hand, the CLV0.5 treated rabbits showed a significant decrease in lymphocytes (LYM) % and MCV compared to the control group.

### 3.3. Blood Biochemical Parameters

The influences of the dietary CLV on the serum metabolites of growing male rabbits are revealed in Table 6. Most of the serum parameters were non-significantly different by CLV supplementation in rabbit diets. Meanwhile, alanine aminotransferase (ALT), triglycerides (TG), and very low-density lipoprotein (VLDL) levels were statistically affected by the dietary CLV addition (*p* < 0.05). For the liver enzymes, the dietary supplementation of CLV significantly (*p* < 0.05) decreased the serum activity of ALT compared to the basal diet; the lowest value of ALT was noticed in CVL1.5 group, while the highest one was detected in the control group (Table 6). The levels of serum TG were reduced (*p* < 0.05) in the CLV supplemented groups compared to the control. Enriching a growing rabbit diet with CLV0.5 and CLV1.0 significantly declined (*p* < 0.05) the serum content of VLDL in comparison with the CLV1.5 group and the control (Table 6).

### 3.4. Immunity and Oxidative Status

The dietary supplementation of CLV to growing rabbit diets resulted in a significant increase in IgG and Ig M concentrations (Table 7). Specifically, the addition 0.5 or 1.5 g CLV/kg to the rabbit diet enhanced the serum values of IgG. Meanwhile, the addition of CLV at levels 0.5, 1.0, and 1.5 g/kg diet significantly (*p* < 0.05) enhanced IgM compared to the control group. In a converse trend, supplementing growing rabbit diets with CLV reduced the serum levels of malondialdehyde (MDA) compared to the control. The levels of 0.5 or 1.0 g CLV/kg diet exhibited the best activities of glutathione compared to those of the other groups. No significant changes were detected in the activities of SOD and TAC between the treated and control groups.

## 4. Discussion

In the present study, the supplementation of CLV (0.5, 1, or 1.5 g/kg diet) to the rabbit feed did not statistically affect their growth performance (FI, FCR, BWG, CBWG, and carcass traits). The non-significant impacts of CLV on body weight gain (BWG), CBWG, FI, and FCR between all treated groups and the control are supported by Kotrbáček et al. [8], who found that the addition of 0.5% CLV biomass to broiler diets had no influence on the BWG. Our results are consistent with the findings of Oh et al. [17]. Additionally, Kotrbáček et al. [18] postulated that adding 0.5% CLV to broiler diets had no influence on the growth performance of broiler chicks [11]. Conversely, Kang et al. [7] stated that numerous chlorella-based supplements counting dried chlorella powder (DCP), liquid media, or chlorella growth factor (CGF) supplemented to broiler chicks diets boosted BWG, but did not affect FI and FCR. Several previous results indicated that adding CLV in different forms to broiler diets had an affirmative influence on the BWG of chicken because of the presence of some constituents that enhanced nutrients’ digestibility, which might exhibit better BWG and decrease the total feed cost [19,20]. It could be suggested that this inconsistency among the reported literature might be related to a diversity of sources of chlorella-based products, including powdered and liquid chlorella, chlorella extracts, and fermented chlorella and their levels, which have been used or introduced as a feed additive in different animal species.

In the current work, the results in Table 4 exhibited that all carcass parameters and relative organ weights (such as giblets, heart, kidney, lung, liver and spleen) were not significantly influenced by CLV addition. Our results are in agreement with An et al. [9], who reported that there were no significant differences in all the carcass parameters of broilers affected by dietary CLV inclusion. Moreover, Choi et al. [20] confirmed that the supplementation of recombinant CLV did not affect all the carcass traits of broilers except for breast muscle weight and drip loss.

The blood hematology evidences were within the reference range, and no signs of deleterious influences were monitored. Our results revealed that the addition of 0.5 g CLV to the basal diet resulted in a significant increase in PLT, HCT, MCH, and MCHC values, and a reduction in LYM and MCV % (Table 5). In previous studies, it has been shown that dietary phytogenic in rabbit diets as an immune stimulator agent had a significantly lower erythrogram compared to those in the control group [1,3]. Conversely, Choi et al. [20] reported that the use of CLV in chicken feed did not have a beneficial effect on the erythrogram counts. Furthermore, Kang et al. [7] suggested that broilers fed a diet enriched with 1% fresh liquid CLV had higher white blood cell counts and lymphocytes compared with the control group. Such fluctuations in the levels of hematobiochemical variables in growing rabbit blood may be attributed to the differences in metabolic processes due to CLV supplements.

With regard to effect of CLV on total protein, albumin, and globulins levels, no significant differences could be found in this study among treated groups and the control. The CLV treatments reduced the liver function enzymes in the growing rabbit blood plasma. Our outcomes related to the serum ALT level displayed that the levels of CLV were safe and useful in expressing liver function. The reduced lipid profile with respect to total glycerides and VLDL levels in the growing rabbits diets supplemented with CLV is interesting. In accordance with our findings, Choi et al. [20] displayed that broilers fed with CLV had decreased (*p* < 0.05) lipid profiles in the serum in total cholesterol and LDL levels compared to those in the control [21,22]. We suggested that this finding can be explained by the plausible decline in acetyl–CoA enzyme fusion that is essential for the biosynthesis of fatty acids.

Chlorella species is considered as having antimicrobial, anti-inflammatory, immunomodulatory, and analgesic activities, or even being an anticancer agent [23,24,25]. In addition, CLV has an efficient supply to promote the immunity status of animals, and has a remarkable feature for improving the health and welfare of animals [5,26]. The addition of CLV at 0.5 and 1.5 g/kg diet in rabbit diet resulted in a significant increase (*p <* 0.05) in IgG levels in rabbit blood plasma. Moreover, different levels CLV increased (*p <* 0.05) IgM levels (Table 7). Similar to our results, Kang et al. [7], An et al. [9], and Kanouchi et al., [27,28] suggested that these consequences are mainly due to high levels of omega-3 PUFAs and antioxidants in CLV that modulated immune responses in rodents by enhancing the production of IgG and IgM [7,9,29]. Numerous types of single-cell green algae, particularly CLV and Spirulina species, have been informed as excellent sources of immunoregulatory functions, such as β carotene, β-glucan, and vitamin B12, which play fundamental functions in immune and inflammatory responses in humans and animals [30,31]. Furthermore, CLV can promote the activity of macrophages and immune cells to increment its synthesis of γ-interferon protein, which has the capability to protect the body cells against infections or pathogens. Hence, CLV can stimulate the capability of the immune system to fight against foreign protein and pathogens. The supplementation of 0.5 and 1 g/kg diet of CLV presented greater levels of GSH and lower contents of MDA in rabbit blood compared to that in the control group. No significant influences have been noted by the CLV addition to growing rabbit diets regarding SOD and total (TAC) contents. In contrast, Tsiplakou et al. [10] reported no significant effects on MDA contents after CLV addition to goat diets. However, the dietary supplementation with CLV caused a significant decrease in MDA content in goat’s milk. These bioactive ingredients of CLV may delay the oxidation of lipids (MDA) or proteins (protein carbonyl) and other nutrients by suppressing the propagation of oxidation reactions. However, there is global increasing attention paid to the screening and production of alternative natural antioxidants or anticancer agents from microalgae [5,32]. The respective response of the CLV on immunity response (IgM and IgG activities) in growing rabbit plasma suggests an improvement in the antioxidant defense system, as well as a further reduction in MDA, which represents the lipid peroxidation [33,34,35,36]. This shows the beneficial health implications of CLV.

## 5. Conclusions

In conclusion, diets supplemented with different levels of CLV up to 1 g/kg diet had respectable effects on some traits studied, especially immune responses, as well as antioxidant and hematobiochemical indices. It could be theorized that using CLV as a natural feed supplement in rabbit diets can boost rabbits’ health. It is consequently a means to provide consumers with a healthy food and to get rid of using antibiotics or drugs as growth promoters during the fattening period.

## Figures and Tables

**Table 1:** Ingredients and composition of the basal diet of growing rabbits (as fed).

Items	Basal Diet
Ingredient	%
Maize	20
Soybean meal	20
Wheat bran	16
Berseem hay	30
Barley grain	10
Molasses	2
Limestone	1
NaCl	0.5
Premix *	0.5
Calculated composition **	%
ME (Metabolizable energy), MJ/kg	7.95
Crude protein	17.50
Calcium	0.88
Available phosphorus	0.20
Analyzed composition	%, on DM (dry matter) basis
Crude protein	16.54
Ether extract	2.25
Crude fiber	12.33
Organic matter	90.57
Ash	9.43
Nitrogen-free extract	59.45

* Each 1 kg of premix (minerals and vitamins mixture) contains vit. A, 20,000 IU; vit. D3, 15,000 IU; vit. E, 8.33 g; vit. K, 0.33 g; vit. B1, 0.33 g; vit. B2, 1.0 g; vit. B6, 0.33 g; vit. B5, 8.33 g; vit. B12, 1.7 mg; pantothenic acid, 3.33 g; biotin, 33 mg; folic acid, 0.83 g; choline chloride, 200 g. ** Calculated according to NRC [14].

**Table 2:** Live body weight and body weight gain of growing New Zealand white (NZW) rabbits as affected by dietary treatments at 13 weeks of age.

Items	Live Body Weight (g)			Cumulative Body Weight Gain (g)		
	5 Weeks	9 Weeks	13 Weeks	5–9 Weeks	9–13 Weeks	5–13 Weeks
Control	675.00	1288.33	2141.67	613.33	853.33	1466.67
CLV0.5	663.33	1298.33	2193.33	635.00	895.00	1530.00
CLV1.0	658.33	1315.00	2175.00	656.67	860.00	1516.67
CLV1.5	673.33	1296.67	2101.67	623.33	805.00	1428.33
SEM	9.36	8.52	24.94	8.13	23.22	25.05
*p* value	0.934	0.787	0.646	0.290	0.656	0.512

CLV0.5: basal diet + 0.5 g chlorella powder/kg diet; CLV1.0: basal diet + 1.0 g chlorella powder/kg diet; CLV1.5: basal diet + 1.5 g chlorella powder/kg diet. CLV: *Chlorella vulgaris*; SEM: Standard error of mean. Means in the same column with no superscript letters after them or with a common superscript letter following them are not significantly different (*p* < 0.05).

**Table 3:** Feed intake and feed conversion ratio of growing NZW rabbits as affected by dietary treatments at 13 weeks of age.

Items	Feed Intake (g)			Feed Conversion Ratio (g Feed/g Gain)		
	5 Weeks	9 Weeks	13 Weeks	5–9 Weeks	9–13 Weeks	5–13 Weeks
Control	69.17	115.67	92.42	3.95	4.76	4.35
CLV0.5	67.92	114.67	91.29	3.74	4.49	4.12
CLV1.0	67.92	119.00	93.46	3.63	4.89	4.26
CLV1.5	65.42	110.33	87.88	3.68	4.83	4.25
SEM	1.67	2.77	1.58	0.10	0.14	0.06
*p* value	0.913	0.796	0.685	0.731	0.824	0.616

CLV0.5: basal diet + 0.5 g chlorella powder/kg diet; CLV1.0: basal diet + 1.0 g chlorella powder/kg diet; CLV1.5: basal diet + 1.5 g chlorella powder/kg diet. CLV: *Chlorella vulgaris*. SEM: Standard error of mean. Means in the same column with no superscript letters after them or with a common superscript letter following them are not significantly different (*p* < 0.05).

**Table 4:** Carcass traits of growing NZW rabbits as affected by dietary treatments at 13 weeks of age.

Items	Carcass Traits (as % of Pre-Slaughter Weight)							
	Carcass	Dressing	Giblets	Heart	Kidney	Lung	Liver	Spleen
Control	55.25	57.45	6.64	3.50	8.92	9.25	44.35	0.36
CLV0.5	56.12	58.27	6.43	3.03	9.46	8.77	42.68	0.32
CLV1.0	52.73	54.86	6.34	2.98	9.12	8.69	42.17	0.45
CLV1.5	56.39	58.53	6.44	3.08	9.29	8.51	43.13	0.45
SEM	0.84	0.85	0.15	0.10	0.22	0.20	1.19	0.02
*p* value	0.445	0.456	0.941	0.269	0.877	0.669	0.949	0.100

CLV0.5: basal diet + 0.5 g chlorella powder/kg diet; CLV1.0: basal diet + 1.0 g chlorella powder/kg diet; CLV1.5: basal diet + 1.5 g chlorella powder/kg diet; CLV: *Chlorella vulgaris*. SEM: Standard error of mean. Means in the same column with no superscript letters after them or with a common superscript letter following them are not significantly different (*p* < 0.05).

**Table 5:** Hematology of growing NZW rabbits as affected by dietary treatments at 13 weeks of age.

Items	HGB (g/dL)	RBCs (10^6^/μL)	WBCs (10^3^/μL)	PLT (10^3^/μL)	HCT (%)	BASO (%)	ESIN (%)	LYM (%)	MON (%)	MCV (µm^3^)	MCH (pg)	MCHC (g/dL)
Control	12.92	4.58	3.87 ^b^	875.33 ^b^	40.19 ^a,b^	0.51	1.62	50.67 ^a^	0.36 ^a,b^	91.90 ^a^	27.03 ^b^	31.31 ^b^
CLV0.5	13.02	5.16	3.77 ^b^	1119.67 ^a^	41.36 ^a^	0.49	1.29	37.65b ^c^	0.30 ^b^	80.86 ^b^	31.82 ^a^	38.64 ^a^
CLV1.0	13.07	4.66	4.25 ^b^	841.00 ^b^	38.69 ^b^	0.40	1.30	34.93 ^c^	0.35 ^a,b^	89.21 ^a^	28.79 ^b^	32.22 ^b^
CLV1.5	12.70	4.64	5.15 ^a^	853.67 ^b^	38.58 ^b^	0.45	1.25	45.70 ^a,b^	0.41 ^a^	87.11 ^a^	27.49 ^b^	31.36 ^b^
SEM	0.14	0.13	0.20	13.59	0.42	0.02	0.07	2.23	0.01	1.44	0.65	0.95
*p* value	0.848	0.445	0.018	0.040	0.027	0.199	0.184	0.013	0.037	0.012	0.010	0.001

CLV0.5: basal diet + 0.5 g chlorella powder/kg diet; CLV1.0: basal diet + 1.0 g chlorella powder/kg diet; CLV1.5: basal diet + 1.5 g chlorella powder/kg diet. CLV: *Chlorella vulgaris*. HGB: hemoglobin; RBCs: red blood cells; WBCs: white blood cells; PLT: platelet count; HCT: hematocrit; BASO: basophils; ESIN: eosinophils; LYM: lymphocytes; MON: monocytes; MCV: mean corpuscular volume; MCH: mean corpuscular hemoglobin; MCHC: mean corpuscular hemoglobin concentration. SEM: Standard error of mean. ^a,b,c^ Means in the same column with different superscript letter following them are significantly different (*p* < 0.05).

**Table 6:** Profiles of growing NZW rabbits as affected by dietary treatments at 13 weeks of age.

Items	TP (g/dL)	Alb (g/dL)	Glob (g/dL)	A/G (%)	AST (IU/L)	ALT (IU/L)	TBil (mg/dL)	DBil (mg/dL)	Uric Acid (mg/dL)	TC (mg/dL)	TG (mg/dL)	HDL (mg/dL)	LDL (mg/dL)	VLDL (mg/dL)	LDH (U/L)
Control	6.51	4.38	2.13	2.21	22.98	81.15 ^a^	0.74	0.19	1.66	112.01	111.52 ^a^	51.26	35.17	28.21 ^a^	155.10
CLV0.5	6.65	3.71	2.94	1.30	22.78	71.82 ^a,b^	0.84	0.19	1.41	124.66	90.78 ^b^	50.86	33.35	18.48 ^b^	143.55
CLV1.0	6.59	4.44	2.15	2.10	21.41	70.20 ^a,b^	0.80	0.18	1.52	110.54	84.05 ^c^	48.98	28.39	18.21 ^b^	146.28
CLV1.5	6.79	4.53	2.26	2.14	21.83	65.05 ^b^	0.81	0.18	1.55	104.86	83.23 ^c^	49.79	32.30	22.24 ^a,b^	169.72
SEM	0.15	0.21	0.18	0.20	0.58	2.46	0.02	0.01	0.04	4.08	5.054	0.95	1.39	1.70	5.85
*p* value	0.950	0.547	0.340	0.358	0.792	0.013	0.577	0.654	0.473	0.249	0.002	0.417	0.869	0.040	0.106

CLV0.5: basal diet + 0.5 g chlorella powder/kg diet; CLV1.0: basal diet + 1.0 g chlorella powder/kg diet; CLV1.5: basal diet + 1.5 g chlorella powder/kg diet. CLV: *Chlorella vulgaris.* TP: total protein; Alb: albumin; Glob: globulin; A/G: albumin/globulin ratio; AST: aspartate aminotransferase; ALT: alanine aminotransferase; TBil: total bilirubin; DBil: direct bilirubin; TC: total cholesterol; TG: triglycerides; HDL: high-density lipoprotein; LDL: low-density lipoprotein; VLDL: very low-density lipoprotein; LDH: lactate dehydrogenase. SEM: Standard error of mean. ^a,b,c^ Means in the same column with different superscript letter following them are significantly different (*p* < 0.05).

**Table 7:** Immunity and oxidative status of growing NZW rabbits as affected by dietary treatments at 13 weeks of age.

Items	Immunity Parameters		Oxidative Status			
	IgG (mg/dL)	IgM (mg/dL)	SOD (U/mL)	MDA (nmol/mL)	GSH (ng/mL)	TAC (ng/mL)
Control	48.82 ^b^	72.83 ^c^	0.20	0.22 ^a^	0.20 ^c^	0.20
CLV0.5	53.46 ^a^	75.56 ^a^	0.20	0.19 ^b^	0.22 ^a^	0.21
CLV1.0	52.53 ^a,b^	75.74 ^a^	0.21	0.19 ^b^	0.22 ^a^	0.22
CLV1.5	53.06 ^a^	73.73 ^b^	0.21	0.20 ^b^	0.21 ^b^	0.21
SEM	0.67	0.88	0.00	0.01	0.00	0.01
*p* value	0.034	0.039	0.561	0.026	0.004	0.648

CLV0.5: basal diet + 0.5 g chlorella powder/kg diet; CLV1.0: basal diet + 1.0 g chlorella powder/kg diet; CLV1.5: basal diet + 1.5 g chlorella powder/kg diet. CLV: *Chlorella vulgaris*. IgG: immunoglobulin G: IgM: immunoglobulin M; SOD: superoxide dismutase; MDA: malondialdehyde; GSH: glutathione; TAC: total antioxidant capacity. SEM: Standard error of mean. ^a,b,c^ Means in the same column with different superscript letter following them are significantly different (*p* < 0.05).
